# Prescription Pattern of Tofacitinib for Alopecia Areata Among the Dermatologists in Saudi Arabia: A Cross-Sectional Study

**DOI:** 10.7759/cureus.40445

**Published:** 2023-06-15

**Authors:** Abdulaziz S Alsuhibani, Raghad M Alharthi, Saba AlSuhaymi, Muhannad A Alnahdi, Mohammad Almohideb

**Affiliations:** 1 Collage of Medicine, King Saud Bin Abdulaziz University for Health Sciences College of Medicine, Riyadh, SAU; 2 Department of Dermatology, King Abdulaziz Medical City, Riyadh, SAU; 3 Department of Dermatology, King Faisal Specialist Hospital and Research Centre, Riyadh, SAU; 4 Department of Ophthalmology and Vision Science, University of Toronto, Toronto, CAN

**Keywords:** dermatologists, areata, jak inhibitor, kingdom of saudi arabia (ksa), alopecia

## Abstract

Introduction

Alopecia areata (AA) is a complex autoimmune condition that causes nonscarring hair loss. In Saudi Arabia, AA accounts for 1-2% of new dermatological outpatient visits. It typically presents with sharply demarcated round patches of hair loss and may present at any age. Traditional medical therapies include corticosteroids and immunotherapy. Choosing the ideal treatment depends on multiple factors such as patient age, disease severity, efficacy, side effects, and remission rate. Recent medications that have been used for treating AA are Janus kinase inhibitors.

Aim

The aim of the study is to assess the awareness and attitude of dermatologists and their use of Tofacitinib in treating AA.

Method

A cross-sectional study was conducted in 2019 across 14 major cities in Saudi Arabia. A self-administered online questionnaire was specifically developed and used. Dermatologists from government hospitals and private clinics were included through non-probability convenience sampling. The collected data was entered into Microsoft Excel and analyzed using SPSS program version 24.

Results

In total, out of 546 Dermatologists across Saudi Arabia who responded to the questionnaire, 127 (23.2%) physicians prescribed Tofacitinib in their practice. Out of those who prescribed the drug for AA cases, 58 dermatologists (45.6%) prescribed Tofacitinib after the failure of steroid injections. Among the 127 dermatologists who have utilized Tofacitinib in their practice, 92 (72.4%) believe that Tofacitinib is effective in treating AA. Almost 200 (47.7%) Dermatologists who never prescribed Tofacitinib reported that the main reason was due to the unavailability of the drug in the clinic they were practicing.

Conclusions

To conclude, out of 546 dermatologists working in Saudi Arabia, 127 (23.2%) prescribe Tofacitinib to treat AA. Ninety-two (72.4%) of the participants reported the effectiveness of Tofacitinib. Two hundred (47.7%) dermatologists who never prescribe Tofacitinib reported that the main reason was due to the unavailability. However, this would raise the need for more research regarding JAK inhibitors generally and Tofacitinib specifically, focusing on the effectiveness versus the side effects of Tofacitinib.

## Introduction

Alopecia areata (AA) is a complex autoimmune condition that causes nonscarring hair [1]. In Saudi Arabia, AA accounts for 1-2% of new dermatological outpatient visits [2]. A systemic review indicated a similar worldwide lifetime incidence of around 2% [3]. It typically presents with sharply demarcated round patches of hair loss and may present at any age. AA has a variable clinical course with spontaneous remission, followed by exacerbations, or relentless progression to involve the entire scalp (alopecia totalis) and, uncommonly, all body hair (alopecia universalis; AU).

AA has an unpredictable prognosis and hair loss may spontaneously remit, although the timeframe for regrowth may be months to years [4]. There is a genetic predisposition to alopecia areata. About 20% of people with alopecia areata have a family history. The cause and mechanism of alopecia areata development are yet to be completely understood. However, genetic predisposition, immune system autoreactivity, and environmental precipitators are aspects that identify several processes and factors that have been proven to be major contributors to disease pathophysiology. AA is considered an autoimmune disease with a complex genetic component. Numerous gene mutations and polymorphisms have been identified to increase susceptibility to developing the disease; as well as other inflammatory and autoimmune diseases. Traditional medical therapies include corticosteroids, immunotherapy, and light therapy. Systemic medications such as corticosteroids, cyclosporine, and methotrexate have been used for severe forms but had many limitations including side effects and a high relapse rate. Recent medications that have been used for treating AA include Janus kinase inhibitors. These have been approved for treating other immune-mediated & inflammatory diseases such as rheumatoid arthritis, myelofibrosis, and psoriatic arthritis by blocking the JAK inflammatory pathway that involves IL2, IL5, and IFN-γ. Oral and topical JAK inhibitor treatments have both prevented and reversed AA in mouse models. There have been several case series and reports demonstrating hair regrowth in patients with AA and AU. Moreover, many clinical trials are ongoing for AA involving JAK inhibitors such as ruxolitinib, tofacitinib, and baricitinib [5].

In this study, we seek to explore the awareness of dermatologists from multiple governmental and private hospitals in Saudi Arabia, aiming to assess their knowledge regarding the use and prescription of Tofacitinib for treating AA.

## Materials and methods

An observational cross-sectional study was conducted from October 2019 to March 2020 across 14 major cities in Saudi Arabia. The targeted areas are dermatology departments at both government hospitals and private clinics in Jeddah, Mecca, Al Taif, Abha, Jazan, Najran, Al Jouf, Tabuk, Hail, Al Dammam, Al Khobar, Al Ahsa, and Al Qatif. A self-administered online questionnaire was developed and collected using an electronic survey. The electronic survey was made using Google Forms software by the research team and distributed to all dermatologists. However, medical students and interns were excluded from the study. The questionnaire was validated through a pilot study on 25 doctors before distributing it. The total number of participants was 546 dermatologists. The collected data was entered into Microsoft Excel, exported, and then analyzed using SPSS program version 24. Categorical variables (e.g. gender) will be presented as percentages and frequencies. On the other hand, numerical variables (e.g. years of experience) will be presented by mean and standard deviation. The Chi-square test was used to compare categorical variables, and the ANOVA test was utilized for any numerical associations. A p-value of <0.05 indicated a statistically significant association. 

## Results

A total number of 546 responses were obtained from dermatologists practicing across the kingdom of Saudi Arabia (Riyadh, Jeddah, Dammam, Alahsa, Alkhobar, Alqatif, Makkah, Taif, Abha, Jazan, Najran, Aljouf, Ha’il, Tabuk). In total, dermatologists who used Tofacitinib for treating AA comprised 127 (23.3%) physicians, and 419 (76.7%) reported not having used Tofacitinib for treating AA (Table 1), while only 11 (2%) dermatologists reported using a different type of JAK inhibitor (Table 2).

**Table 1:** Dermatologists prescribing Tofacitinib for alopecia areata

	Frequency	%
No	419	76.70%
Yes	127	23.30%
Total	546	100%

**Table 2:** Dermatologists prescribing JAK inhibitors for alopecia areata

	Frequency	%
No	535	98%
Yes	11	2%
Total	546	100%

Dermatologists who have used Tofacitinib reported the use of other medications in combination with Tofacitinib such as topical steroids by 67 (12.3%) dermatologists, and topical Minoxidil by 67 (12.3%) dermatologists (Table 3).

**Table 3:** Medications used in combination with Tofacitinib

	Count	%
Topical Steroids	67	12.30%
Topical Minoxidil	67	12.30%
Steroid injections	51	9.30%
Zinc	21	3.80%
Systemic Steroids	13	2.40%
DPCP	13	2.40%
Anthralin	10	1.80%
Topical Calcipitriol	9	1.60%
Methotrexate	8	1.50%
Minoxidil PO	8	1.50%
Dupilumab (Dupixent)	6	1.10%
Cryotherapy	4	0.70%
Cyclosporine	1	0.20%

A total of 294 dermatologists prescribed Tofacitinib at varying steps in the management plan. Fifty-eight dermatologists (10.7%) prescribed Tofacitinib after the failure of other medications like steroid injections, 45 (8.2%) after the failure of topical steroids, 42 (7.7%) after the failure of topical Minoxidil, and 41 (7.5%) after the failure of systemic steroids. Eleven (2%) dermatologists prescribed Tofacitinib as the first-line treatment (Table 4). 

**Table 4:** Failure of other treatments

	Count		%
Steroid Injections		58	10.70%
Topical Steroids		45	8.20%
Topical Minoxidil		42	7.70%
Systemic Steroids		41	7.50%
Methotrexate		26	4.80%
Diphenylcyclopropenone (DPCP)		22	4.00%
Anthralin		22	4.00%
Cyclosporine		14	2.60%
As first line		11	2.00%
Topical Calcipitriol		5	0.90%
Zinc		3	0.50%
Minoxidil PO		3	0.50%
Dupilumab (Dupixent)		1	0.20%
Cryotherapy		1	0.20%

Alopecia totalis was the most common type of AA that Tofacitinib was prescribed for (Table 5).

**Table 5:** Tofacitinib prescribed based on the type of alopecia areata

	Count	%
Totalis	94	17.20%
Universalis	89	16.30%
Ophiasis	21	3.80%
Patchy	16	2.90%
Sisaipho	12	2.20%
Alopecia Incognita	7	1.30%

Patients’ age also seemed a contributing factor when prescribing Tofacitinib, where 104 (19%) dermatologists prescribed the medication to only adults (20 years and older) (Table 6). Dermatologists were also asked about encountering any limitations when prescribing Tofacitinib. Limitations were sorted into three categories: hospital-related, drug-related, and patient-related limitations. The most frequent limitations encountered by dermatologists were hospital-related including the medication's non-availability (90 (16.5%) dermatologists), and insurance non-coverage of the medication (38 (7%) dermatologists) where not having sufficient evidence regarding the drug’s use was the most encountered drug-related limitation (24 (4.4%) dermatologists). Finally, patient-related limitations reported the presence of contraindications (17 (3.1%) dermatologists) as the highest (Table 7). Thirt-four (6.2%) dermatologists encountered patients suffering from Tofacitinib side effects like headache, nausea, and respiratory tract infections reported by 20 (3.7%), 14 (2.6%), and seven (1.3%) dermatologists, respectively (Table 8).

**Table 6:** Tofacitinib prescribed based on age group

	Count	%
Adults (20 years of age and older)	104	19.00%
Adolescents (12-19 years of age)	35	6.40%
All age groups	10	1.80%
Pediatrics	4	0.70%

**Table 7:** Limitations for prescribing Tofacitinib

	Count	%
The medication is not available	90	16.50%
Insurance non-coverage of the medication	38	7.00%
There are not enough studies or clinical trials	24	4.40%
Contraindications	17	3.10%
Relatively new	15	2.70%
Patient refusal	12	2.20%
Side-effects	6	1.10%

**Table 8:** Side effects of Tofacitinib use

	Count	%
Have you noticed any side effects of Tofacitinib?	34	6.20%
Headache	20	3.70%
Nausea	14	2.60%
Respiratory tract infections	7	1.30%
Raised LDL	7	1.30%
Anaemia	4	0.70%
Raised liver transaminase	3	0.50%
Urinary tract infections	2	0.40%
Viral gastroenteritis	2	0.40%
Raised creatinine	2	0.40%
Neutropenia	2	0.40%
Varicella zoster	2	0.40%
Raised HDL	1	0.20%
Paronychia	0	0.00%
Thrombocytopenia	0	0.00%

Dermatologists reported various reasons for not having prescribed Tofacitinib in their practice. The reasons were divided into three categories: hospital-related, drug-related, and patient-related reasons. Medication unavailability was reported by 199 (36.4%) dermatologists, while 56 (10.3%) dermatologists reported the reason for not prescribing was due to a lack of drug awareness (Table 9). A total of 329 (60.3%) dermatologists reported topical steroids as the most common modality for the treatment of AA in their patients (Table 10).

**Table 9:** Dermatologists' limitations for prescribing Tofacitinib

	Count	%
The medication is not available	199	36.40%
Do not know the drug	56	10.30%
Relatively new	43	7.90%
There are not enough studies or clinical trials	41	7.50%
Insurance non-coverage of the medication	39	7.10%
Side-effects	22	4.00%
Not FDA approved	16	2.90%
Contraindications	12	2.20%
Patient refusal	9	1.60%

**Table 10:** Treatments used to treat alopecia areata

	Count	%
Topical Steroids	329	60.30%
Steroid injections	269	49.30%
Topical Minoxidil	243	44.50%
Systemic Steroids	142	26.00%
Zinc	83	15.20%
DPCP	65	11.90%
Anthralin	54	9.90%
Topical Calcipitriol	52	9.50%
Methotrexate	46	8.40%
Cyclosporine	26	4.80%
Dupilumab (Dupixent)	17	3.10%
Cryotherapy	15	2.70%
Minoxidil PO	13	2.40%

Among all 546 dermatologists, 47 (8.6%) agreed that Tofacitinib is safe in general, while 424 (77.7%) dermatologists did not agree that Tofacitinib is safe (Table 11). Of all dermatologists, 209 (38.3%) would consider prescribing Tofacitinib for AA, while 337 (61.7%) did not consider prescribing it (Table 12). Among the 127 dermatologists who have utilized Tofacitinib in their practice, 92 (72.4%) agreed, and only one (0.8%) disagreed that Tofacitinib is effective in treating AA (Table 13). Among these 127 dermatologists, 71 (55.9%) agreed that Tofacitinib is more effective in treating AA than the other treatment options (Table 14).

**Table 11:** Dermatologists' perception of the safety of Tofacitinib

	Frequency	%
No	424	77.70%
Relatively safe	67	12.30%
Yes, safe in general	47	8.60%
I don’t know	8	1.50%

**Table 12:** Dermatologists' consideration to prescribe Tofacitinib

	Frequency	%
No	337	61.7%
Yes	209	38.3%
Total	546	100%

**Table 13:** Effectiveness of Tofacitinib in treating alopecia areata

	Frequency	%
Agree	92	72.4%
Disagree	1	0.8%
Total	127	100%

**Table 14:** Effectiveness of Tofacitinib in treating alopecia areata in comparison to other treatments

	Frequency	%
Agree	71	55.9%
Neutral	54	42.5%
Disagree	2	1.6%
Total	127	100%

A total of 283 (51.8%) dermatologists were female and 275 (50.4%) dermatologists were Saudi. The position title of dermatologists varied with consultants being the most common position (29.5%). The number of experience years also varied with one to three years of experience accounting for 184 (35%) consultants (Table 15).

**Table 15:** Demographic characteristics

		Frequency	%
Gender	male	263	48.20%
	female	283	51.80%
	Total	546	100%
Nationality	Saudi	275	50.40%
	Non-Saudi	271	49.60%
	Total	546	100%
Position title	Resident	145	26.60%
	Specialist	240	44%
	Consultant	161	29.50%
	Total	546	100%
Consultant years of experience	1-3 years	184	35%
	3-5 years	110	21%
	5-10 years	110	21%
	More than 10 years	121	23%
	Total	525	100%

The fact that dermatologists prescribed Tofacitinib in their practice was cross-matched with their characteristics. There was no statistically significant relation between prescribing Tofacitinib and the reported years of experience as consultants, with a p-value of 0.505. On the other hand, there was a statistically significant association between prescribing Tofacitinib and the gender of dermatologists, with a p-value of 0.000; that is, Tofacitinib was prescribed more by male dermatologists. A statistically significant relationship was found between prescribing Tofacitinib and the nationality (Saudi) of the dermatologist and their position title (residents), with both p-values of 0.000 (Table 16).

**Table 16:** Pearson Chi-Square tests for demographic characteristics Results are based on nonempty rows and columns in each innermost subtable. * The Chi-square statistic is significant at the .05 level.

Pearson Chi-Square Tests		
Gender	Chi-square	14.566
	df	1
	Sig.	.000*
Nationality	Chi-square	39.533
	df	1
	Sig.	.000*
Position title	Chi-square	21.256
	df	2
	Sig.	.000*
Consultant years of experience	Chi-square	2.341
	df	3
	Sig.	0.505

## Discussion

Tofacitinib is an oral JAK inhibitor that has shown promising results in treating alopecia areata as with other dermatological diseases [1-4,6]. The use of Tofacitinib and other JAK inhibitors for dermatological diseases, especially for AA, is relatively new and yet to be completely evaluated. Up to our knowledge, studies have not evaluated dermatologists’ utility of Tofacitinib for AA. Therefore, this study aimed to provide insight into the prevalence of Tofacitinib use in treating AA and relevant related factors to its prescription among dermatologists in Saudi Arabia. 

A retrospective study by Liu et al. in 2018 found that 90 patients with severe alopecia areata showed a significant increase in hair regrowth after 6 months of tofacitinib treatment [7]. In another study by Mackay-Wiggan et al. in 2016, 66% of their study population experienced 50% or greater hair regrowth after 3 months of tofacitinib treatment [8]. In another study, a systematic review and meta-analysis was done where multiple journals were chosen along with 1244 studies [9]. Of those 1244 studies, only 12 met their criteria where 346 patients that were diagnosed with alopecia areata were available. Of these 346 patients, 288 patients received oral Tofacitinib while 58 patients had oral Ruxolitinib as the treatment of choice [9]. They showed an overall achievement rate of 66% with the severity of the alopecia tool (SALT) [9]. While these studies show promising results of tofacitinib for alopecia areata, it is worth noting that tofacitinib may cause side effects such as increased risk of infection, gastrointestinal perforations, and changes in cholesterol levels. Further studies are needed to understand better the safety and efficacy of tofacitinib for treating alopecia areata. Nevertheless, dermatologists consider tofacitinib an off-label treatment option for patients with alopecia areata in cases where traditional treatments have failed to produce satisfactory results.

The majority (72.4%) of our respondents were dermatologists who have used Tofacitinib and believe the promising results in treating AA, as they reported agreeably regarding its efficacy in treating AA. However, when asked about their perception of its safety, 77.6% of dermatologists did not agree that Tofacitinib was safe. At the same time, there are studies that show a relatively safe profile of Tofacitinib [2-4]. In a study including rheumatoid arthritis patients, a long-term safety profile was documented for up to 9.5 years [3].

Only 23.2% of our respondent dermatologists utilized Tofacitinib in their practice. The main reported limiting factor of prescribing Tofacitinib was drug unavailability, as indicated by nearly half of the dermatologists (47.7%). Similarly, a study across Europe showed the underutilization of synthetic DMARDs, including JAK inhibitors, in treating rheumatoid arthritis was due to financial and administrative constraints [5]. Also, these limiting factors may lead to patterns of nonadherence among patients. In a study that assessed nonadherence to rheumatoid arthritis treatment, Tofacitinib and other biological DMARDs were found to have a primary nonadherence rate of 40.6% [10]. Factors that led to such high figures were sociodemographic variables like the insurance status of patients, age, and medical history [10]. 

## Conclusions

To conclude, out of 546 dermatologists working in Saudi Arabia, 127 (23.2%) prescribe Tofacitinib to treat AA. Ninety-two (72.4%) of the participants reported the effectiveness of Tofacitinib. Two hundred (47.7%) dermatologists who never prescribe Tofacitinib reported that the main reason was due to the unavailability. However, this would raise the need for more research regarding JAK inhibitors generally, and Tofacitinib specifically, focusing on the effectiveness versus the side effects of Tofacitinib.
